# Physiologically based ferritin thresholds to redefine early pregnancy iron screening: a cross-sectional study

**DOI:** 10.1038/s41598-026-54257-x

**Published:** 2026-05-26

**Authors:** Huan Nguyen Pham, Vy Thi Thao Nguyen, Phuc Nguyen Huu Pham, Nghiem Xuan Huynh, Hang Thi Phan, Dung Ngoc Yen Dang, Vinh Thanh Tran, Nien Vinh Lam

**Affiliations:** 1 Hung Vuong Hospital, Ho Chi Minh City, Viet Nam; 2 University of Medicine and Pharmacy at Ho Chi Minh City, Ho Chi Minh City, Viet Nam

**Keywords:** Biomarkers, Diseases, Health care, Medical research, Risk factors

## Abstract

Iron deficiency in early pregnancy is common yet frequently missed because current screening strategies rely on ferritin cutoffs designed to detect iron deficiency anemia. We aimed to establish physiologically based ferritin functional reference limits (FRLs) in healthy, non-anemic pregnant women, specifically to model early iron insufficiency prior to the onset of anemia, where screening strategies are most clinically actionable. The study was conducted at a maternity hospital in Ho Chi Minh City, Vietnam, enrolling first-trimester pregnant women. Participants were included if they had hemoglobin ≥ 11 g/dL, no evidence of infection, body-mass index (BMI) < 30 kg/m², and no confirmed hemoglobinopathies or unexplained microcytosis/hypochromia. The primary outcome was the ferritin FRLs, defined using restricted cubic spline modelling of standalone erythrocyte indices (MCV, MCH, HGB, and RDW). Diagnostic performance was assessed against TSAT. A total of 452 participants had complete data for validation. The final suggested ferritin FRL is 59 ng/mL, which identified more women with non-anemic iron deficiency compared with traditional cutoffs. Validation against TSAT showed excellent rule-out performance with a negative predictive value of 95.8%. A four-zone classification for iron deficiency emerged, including absolute deficiency (< 15 ng/mL), early-stage deficiency (15–26 ng/mL), transitional state (26–59 ng/mL), and physiologic sufficiency (≥ 59 ng/mL). Physiologically derived ferritin thresholds identify early iron deficiency more effectively than conventional cutoffs and provide a grounded framework for screening in the first trimester.

## Introduction

Iron requirements increase markedly during pregnancy to support fetal development and maternal erythropoiesis. While iron deficiency anemia is routinely screened, non-anemic iron deficiency (NAID) remains under-recognized despite its association with maternal fatigue, preterm birth, and impaired neurodevelopment in offspring^1,2^. The traditional ferritin cutoff of 30 ng/mL prioritizes detection of iron deficiency anemia but may fail to capture functional iron deficiency^3^, estimated to range from 14%^4^ to 20.7%^5^, that precedes anemia. Recent studies proposed using functional reference limits (FRLs) derived from physiological relationships between ferritin and erythrocyte indices rather than fixed, pathophysiological thresholds^6,7^. Given the need of evidence to support screening for iron deficiency without anemia^8^, we therefore aimed to define physiologically based ferritin thresholds in early pregnancy using restricted cubic spline modelling of ferritin against erythrocyte indices, and to assess the application of FRLs in screening of iron deficiency versus traditional reference intervals (RIs).

## Materials and methods

### Study design

This study was conducted at Outpatient clinic department, Hung Vuong Hospital, Vietnam. Data were collected from March to August 2025. The study was approved by Ethics Committee of Hung Vuong Hospital, Ho Chi Minh City, Vietnam with approval number CS/HV/24/42. This was an observational cross-sectional study conducted during routine first-trimester antenatal care and no intervention or randomization was performed. The study was registered on ClinicalTrials.gov as an observational study (NCT06990373).

### Participants

Participants were eligible if they had a singleton pregnancy in the first trimester (≤ 13 weeks 6 days), systolic blood pressure < 140 mmHg and diastolic pressure < 90 mmHg, and a body temperature between 35.0 °C and 37.5 °C at the time of examination. To ensure that alterations in erythrocyte indices were driven exclusively by iron status rather than independent genetic or inflammatory confounders, we strictly excluded individuals with evidence of systemic infection or inflammation (defined by clinical signs such as body temperature > 37.5 °C, or positive infectious disease screens for HIV, HBV, HCV, or *Treponema pallidum*). Because genetic polymorphisms such as alpha- and beta-thalassemia are highly endemic in this region and cause microcytosis independent of iron status, we rigorously excluded patients with confirmed hemoglobinopathies on electrophoresis, as well as those presenting with unexplained microcytosis or hypochromia (MCV < 80 fL or MCH < 27 pg) without confirmatory normal electrophoresis results. Furthermore, as the primary objective of this study was to establish thresholds for non-anemic iron deficiency, patients presenting with overt anemia (Hb < 11 g/dL) at baseline were prospectively excluded. All participants provided informed consent.

### Procedures

Between March and August 2025, data were prospectively collected at Hung Vuong Hospital (Ho Chi Minh City, Vietnam) during routine first-trimester antenatal visits (≤ 13 weeks 6 days). Trained staff conducted interviews, physical exams, and drew venous blood following standard protocols. Demographic and clinical data-including age, gestational age, parity, BMI, blood pressure, and temperature-were recorded via patient interviews and electronic records. Laboratory measurements included hemoglobin (HGB), serum iron, ferritin, unsaturated iron-binding capacity (UIBC), total iron-binding capacity (TIBC) calculated from UIBC and serum iron, and calculated transferrin saturation (TSAT). Hemoglobin was measured using DxH 900 automated hematology analyzer, while serum iron, TIBC and ferritin were analyzed using standardized colorimetric, measured on Cobas c502, and chemiluminescent immunoassays, measured on Cobas e801 (Roche Diagnostics, Rotkreuz, Switzerland), in the hospital’s central laboratory. TSAT was derived as the ratio of serum iron to TIBC multiplied by 100. The primary outcome was the determination of functional reference limit and reference intervals (2.5th to 97.5th percentiles) of ferritin. The secondary outcome was the prevalence of non-anemic iron deficiency (NAID), defined as Hb ≥ 11 g/dL in conjunction with traditional cutoff such as ferritin < 30 ng/mL or TSAT < 20% and newly established reference limits.

### Statistical analysis

Data analysis was performed using Jupyter Notebook with pandas, statsmodels, scipy, and matplotlib libraries. Continuous variables were summarized as means ± standard deviations, and categorical variables as counts and percentages. Reference interval (2.5th − 97.5th percentiles) for ferritin were estimated using the non-parametric percentile method following CLSI EP28-A3c guidelines^9^. Outliers (ferritin < 15 ng/mL or > 200 ng/mL; TSAT < 16% or > 45%) were excluded for RI estimation. FRLs were determined by modelling the relationship between serum ferritin and erythrocyte indices (MCV, MCH, RDW, and HGB) via restricted cubic spline regression, excluding individuals with ferritin > 200 ng/mL to prevent skew from iron overload^10^. The FRL was identified as the lowest ferritin value at which the 95% bootstrap confidence interval of the spline’s first derivative encompassed zero across a continuous range, signifying that further increases in ferritin no longer produced measurable changes in erythrocyte indices. Each model was resampled 500 times using non-parametric bootstrapping to obtain robust estimates and 95% confidence intervals.

## Results

**Fig. 1:**
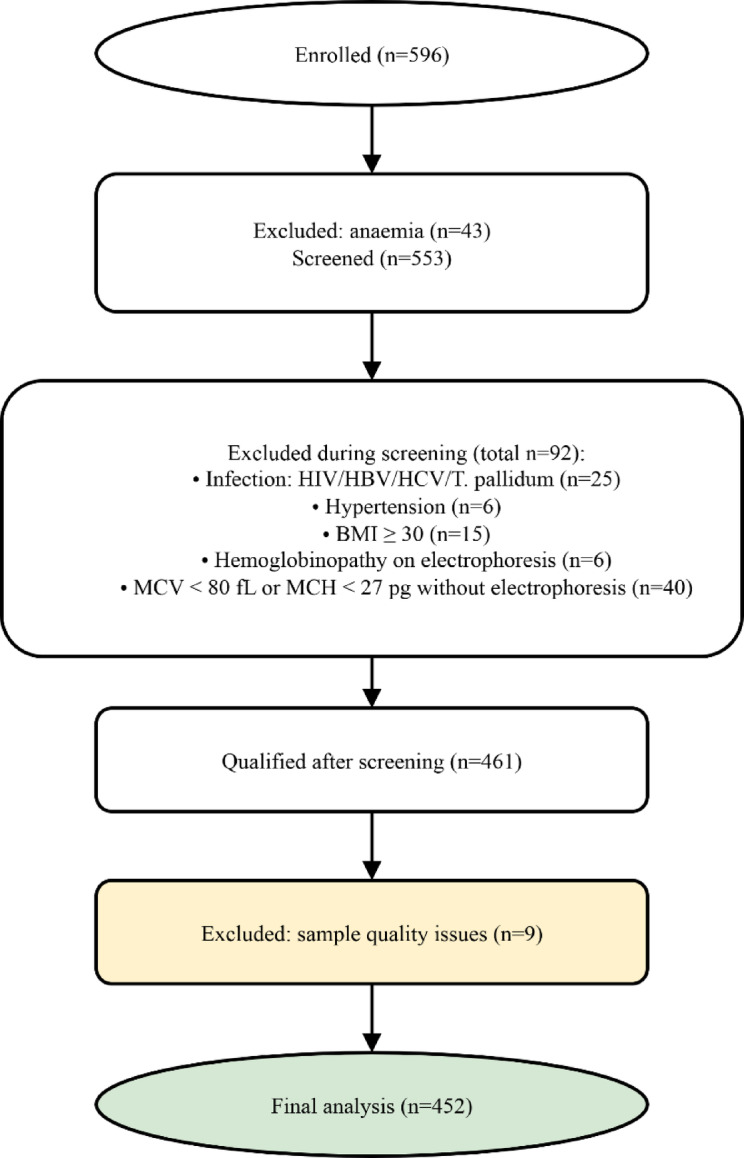
Flow of participant recruitment and exclusion.

Of 596 enrolled participants, 43 with anemia were excluded, leaving 553 for screening. Among these 553, we excluded 25 with infection (HIV, HBV, HCV, or *Treponema pallidum*), 6 with hypertension, 15 with BMI ≥ 30, 6 with hemoglobinopathies on electrophoresis, and 40 with microcytosis/hypochromia (MCV < 80 fL or MCH < 27 pg) without electrophoresis results. After selecting qualified patients, 9 samples failed quality control, yielding a final analytic cohort of 452 patients (Fig. 1).

Participant characteristics are described in Table 1. Overall, a total of 452 first-trimester pregnant patients were enrolled. The mean maternal age was 29.8 ± 5.5 years, and the mean gestational age at enrolment was 11.1 ± 1.7 weeks. Participants had an average weight of 54.2 ± 7.5 kg and height of 156.7 ± 5.4 cm. The mean systolic and diastolic blood pressures were 113.9 ± 9.0 mmHg and 71.0 ± 7.2 mmHg, respectively. Approximately half of the cohort were nulliparous (49.1%), while 50.9% were multigravida. These characteristics reflect a generally healthy and homogeneous group of patients representing early normal pregnancy.

FRLs for ferritin were determined using restricted cubic spline (RCS) models relating ferritin to MCV, MCH, and HGB. Five knots were placed at the 5th, 25th, 50th, 75th, and 95th percentiles of ferritin, following recommended practice for flexible non-linear modelling^11^. Functional reference limits were determined by modeling the relationship between serum ferritin and standalone erythrocyte indices (MCV, MCH, and HGB) via restricted cubic spline regression. Furthermore, we incorporated the modeling of Red Blood Cell Distribution Width (RDW), as an increase in RDW (anisocytosis) is a highly sensitive, early manifestation of iron-restricted erythropoiesis that typically precedes the decline in hemoglobin and MCV^12^. We then estimated the derivative (slope with respect to ferritin) on a fine grid of ferritin values. To account for sampling variability, we performed non-parametric bootstrapping (500 iterations) for internal validation, refitted the spline model in each bootstrap sample, and recomputed the derivative curve. At each ferritin grid point, the 2.5th and 97.5th percentiles of the bootstrapped derivatives defined the 95% confidence interval (CI). The FRL was defined as the lowest ferritin concentration at which this 95% CI of the derivative encompassed zero for a sustained run of points, indicating a plateau of response.

**Table 1:** Patient characteristics.

Baseline characteristics	Mean ± SD
Age (years)	29.81 ± 5.51
Gestational age (weeks)	11.10 ± 1.66
Weight (kg)	54.16 ± 7.49
Height (cm)	156.72 ± 5.37
Systolic blood pressure	113.94 ± 9.03
Diastolic blood pressure	71.00 ± 7.23
Parity	**Counts (percentage)**
Primigravida	222 (49.1%)
Multigravida	230 (50.9%)

**Fig. 2:**
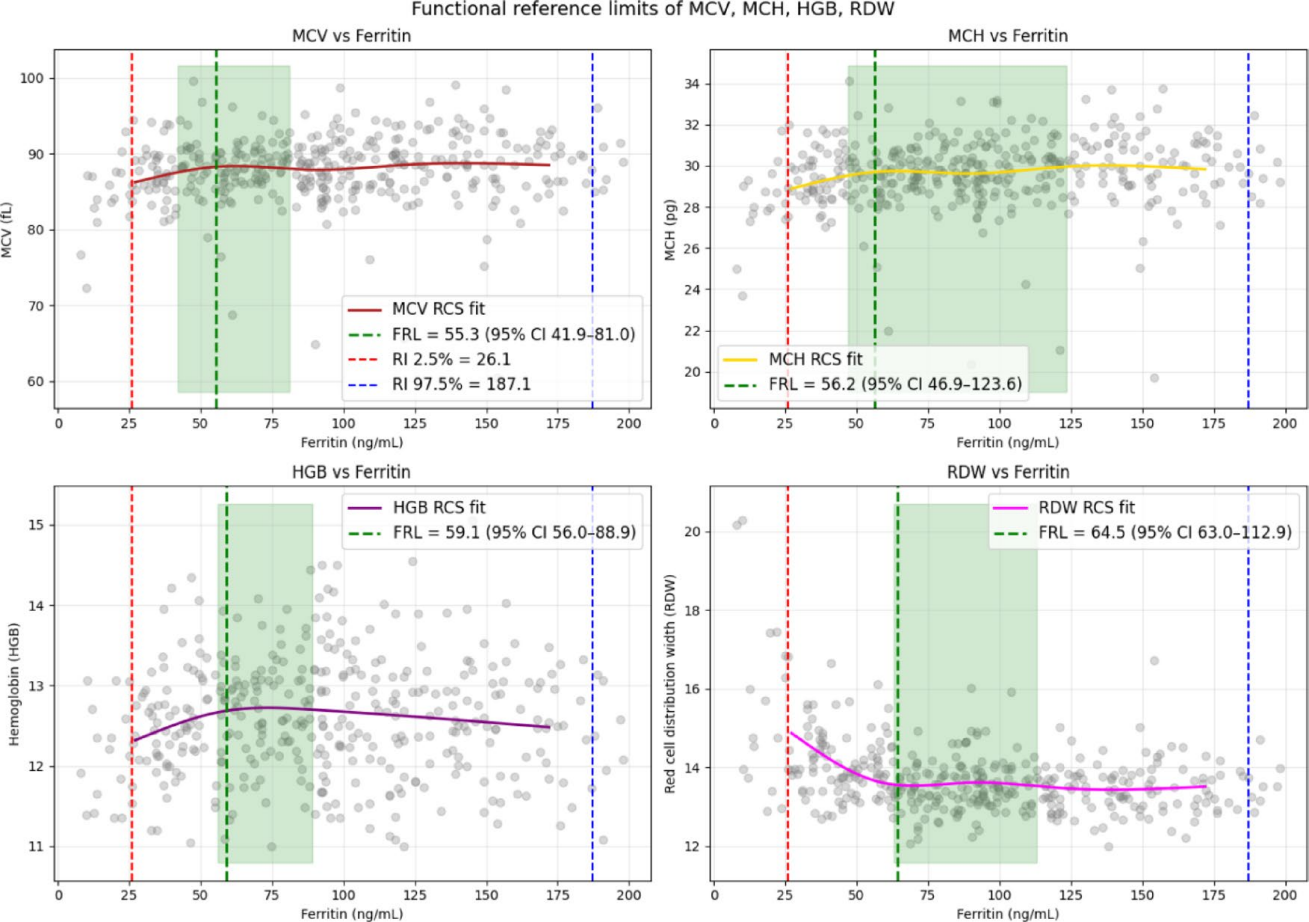
Functional reference limits of MCV, MCH, HGB, RDW.

All four models converged on a ferritin inflection point near 55–60 ng/mL, marking the plateau of erythrocyte indices that reflects physiologic iron adequacy (Fig. 2). For MCV, the functional reference limit (FRL) was estimated at 55.3 ng/mL (95% CI, 41.9–81.0), with the response curve flattening between approximately 40 and 80 ng/mL. The MCH analysis produced a similar FRL of 56.2 ng/mL (95% CI, 46.9–123.6), although the plateau extended over a broader ferritin range, consistent with greater variability at higher concentrations. The HGB analysis produced a FRL of 59.1 with tighter confidence interval (95%CI 56.0–88.9). Modeling of RDW against serum ferritin revealed a distinct inflection point at 64.5 ng/mL (95% CI, 63.0–112.9). Because an increase in RDW is one of the earliest hallmarks of iron-deficient erythropoiesis, it is physiologically consistent that its plateau occurs at a slightly higher ferritin concentration than hemoglobin and MCV. Collectively, all four standalone erythrocyte indices converge on a functional plateau between 55 and 64 ng/mL, robustly validating an overarching clinical threshold around 60 ng/mL for physiological iron sufficiency. For comparison, the reference interval derived from the study indicated a lower limit of 26.1 ng/mL (90% CI, 19.1–33.1) and an upper limit of 187.1 ng/mL (90% CI, 180.2–194.1).

To validate the use of FRL, we tabulated the FRL with TSAT of 16% as the reference definition. Application of the FRL derived from MCV, MCH, HGB, and RDW against TSAT (< 16%) yielded robust classification performance (Table 2). The derived FRLs consistently clustered around the ~ 60 ng/mL mark. For instance, applying the HGB-derived FRL of 59 ng/mL identified 272 true negatives and only 12 false negatives, yielding a high negative predictive value (NPV) of 95.8%. Clinically, this confirms that a ferritin threshold of ~ 60 ng/mL offers strong reassurance for ruling out iron deficiency. The negative predictive value (NPV) was very high (95.8%), indicating that individuals above the FRL threshold were very unlikely to be iron-deficient.

**Table 2:** Diagnostic performance of FRLs in predicting iron deficiency.

Model	*n*	FRL (95% CI)	True Positive	False Positive	True Negative	False Negative	Sens (95% CI)	Specs (95% CI)	PPV (95%CI)	NPV (95%CI)
HGB ~ Ferritin	389	59.1 (56.0–88.9)	20	85	272	12	62.5% (45.3–77.1)	76.2% (71.5–80.3)	19.0% (12.7–27.6)	95.8% (92.8–97.6)
MCV ~ Ferritin	389	55.3 (41.9– 81.0)	17	74	283	15	53.1% (36.4–69.1)	79.3% (74.8–83.2)	18.7% (12.0–27.9)	95.0% (91.9–96.9)
MCH ~ Ferritin	389	56.2 (46.9–123.6)	17	75	282	15	53.1% (36.4–69.1)	79.0% (74.5–82.9)	18.5% (11.9–27.6)	94.9% (91.8–96.9)
RDW ~ Ferritin	389	64.5 (63.0–112.9)	21	102	255	11	65.6% (48.3–79.6)	71.4% (66.5–75.9)	17.1% (11.4–24.7)	95.9% (92.7–97.7)

CI: Confidence interval; FRL: Functional reference limits; Sens: Sensitivity; Specs: Specificity; PPV: Positive predictive value; NPV: Negative predictive value.

**Fig. 3:**
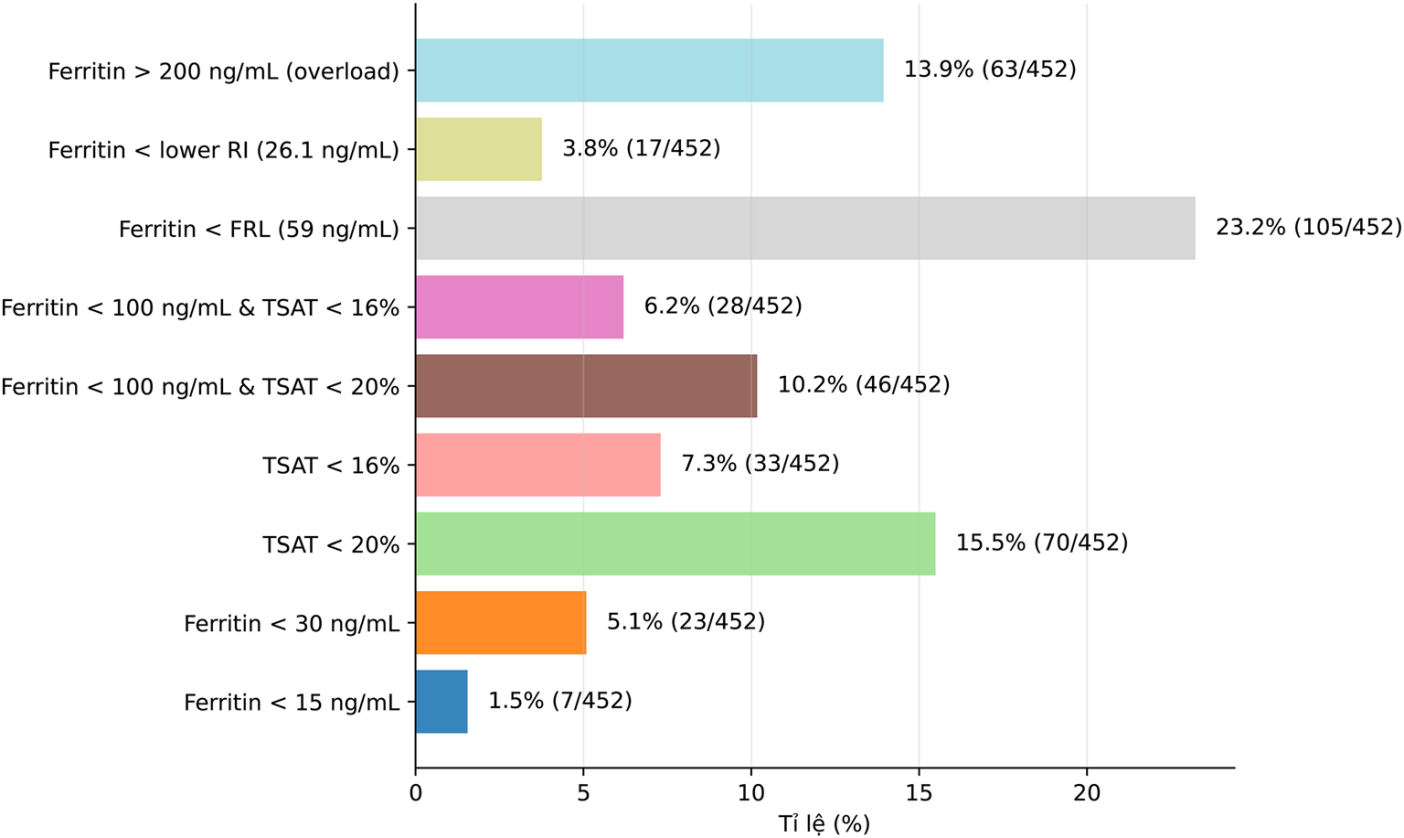
Prevalence of iron overload and non-anemic iron deficiency using different cutoffs.

The prevalence of non-anemic iron deficiency (NAID) varied depending on the applied cutoff criteria (Fig. 3). Using the FRL for ferritin derived from above (59 ng/mL), the prevalence of NAID was 23.2%. Conventional thresholds yielded substantially lower estimates: ferritin < 30 ng/mL identified only 5.1%, and ferritin < 15 ng/mL just 1.5% of the study population. When using transferrin saturation (TSAT) as a cutoff, the prevalence was 7.3% at the < 16% cutoff and 15.5% at the < 20% cutoff. Combining ferritin < 100 ng/mL with TSAT < 16% increased prevalence to 6.2%, while combining ferritin < 100 ng/mL with TSAT < 20% increased prevalence to 10.2%. Overall, the FRL cutoff identified a larger proportion of pregnant patients with iron deficiency compared to traditional ferritin or TSAT thresholds while iron overload also accounts for a substantial proportion of the population (13.9%).

**Fig. 4:**
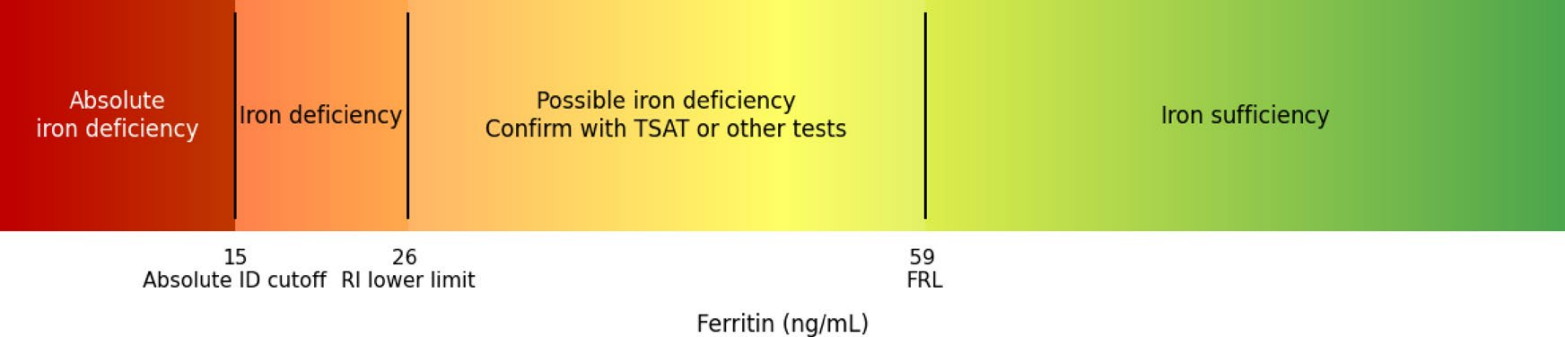
Physiologically based ferritin thresholds for iron status.

Using restricted cubic-spline modeling of hematologic indices (MCV, MCH, and hemoglobin), we identified physiologically distinct ferritin zones that reflect the transition from iron-deficient to iron-sufficient erythropoiesis. Three physiologically distinct thresholds emerged (Fig. 4): the conventional WHO-defined absolute iron-deficiency cutoff at < 15 ng/mL (included as a traditional clinical baseline; while WHO recommends standardizing to the 3rd International Standard (IS)^13^, recovery studies validate the 1st IS-referenced Roche platform’s traceability to the 3rd IS, and regression equations demonstrate a 15 ng/mL reading on this platform corresponds to a highly conservative ~ 12.9 ng/mL on a 3rd IS-calibrated assay by Beckman Coulter analyzer)^14^; the 2.5th percentile lower reference limit statistically derived from our own healthy cohort at ~ 26 ng/mL representing the onset of iron-deficient erythropoiesis; and the physiologically derived FRL at 59 ng/mL, representing the point beyond which iron sufficiency is ensured.

## Discussion

This study defines the first physiologically based ferritin functional reference limit for non-anemic pregnant women, achieved by removing contributors that spuriously increase ferritin concentrations, including infection and obesity^15^. Using red cell indices as biological correlates, we identified a consistent threshold around 55–64 ng/mL at which erythrocyte indices plateau, marking adequate iron supply for erythropoiesis. These values are substantially higher than the traditional ferritin cutoff of 30 ng/mL used in clinical practice or other physiologically based ferritin threshold in studies by Addo OY et al.^7^ or Z.Mei et al.^16^ probably because our study excluded anemic patients and those with hemoglobinopathies. The findings support a re-evaluation of ferritin thresholds for screening, and a threshold to determine iron deficiency in non-anemic patients.

By modelling the observed distribution of erythrocyte indices in a carefully selected non-anemic cohort, our derived FRLs capture the early physiological transition toward iron-restricted erythropoiesis. However, because overtly anemic patients were excluded, the exact mathematical inflection point should be interpreted within this non-anemic screening context. This methodological approach aligns with contemporary understandings of IDE as an active, progressive continuum rather than a binary state^17^. When body iron stores are depleted, erythroid cells actively donate iron to buffer plasma iron levels, manifesting as early microcytosis and hypochromia before progressing to overt anemia. Retaining these patients in the derivation cohort ensured the RCS models could accurately trace this downward physiological trajectory back to its exact point of origin (the inflection point). While the derivation of accurate FRLs requires analyzing this full physiological spectrum, the primary clinical application of our established ≥ 59 ng/mL threshold is to identify IDE in its silent, non-anemic phase. Applying our physiologically derived FRL allows clinicians to evaluate a non-anemic patient and immediately recognize the onset of early IDE long before her hemoglobin drops below 11.0 g/dL.

Our derived lower limit of the ferritin reference interval (~ 26 ng/mL) can be seen as the reflection of the biological onset of functional vulnerability. Recent literature highlights that the conventional WHO threshold of < 15 ng/mL, based largely on expert opinion and radiometric assays from decades ago, identifies a later, more severe stage of iron deficiency. In contrast, Mei et al. recently demonstrated through physiological modeling of hemoglobin and erythrocyte zinc protoporphyrin that the true onset of iron-deficient erythropoiesis begins at ~ 25 µg/L in women^18^. This is further corroborated by their subsequent restricted cubic-spline analysis in US pregnant women, which identified a virtually identical first-trimester functional threshold for iron deficiency of 25.8 µg/L^19^. The remarkable concordance between our internally derived 2.5th percentile limit (~ 26 ng/mL) and these physiologically derived thresholds confirms that patients dropping below 26 ng/mL have exhausted their reserves and are actively entering iron-deficient erythropoiesis (IDE).

Furthermore, our functional modeling demonstrates that true physiological iron sufficiency—the point at which all erythrocyte indices reach a stable plateau—requires a ferritin level of approximately 59 ng/mL. This is consistent with within a broader physiological continuum of iron homeostasis. Isotope studies by Galetti et al.^20^ demonstrated that systemic iron absorption upregulates at a ferritin threshold of ~ 51 ng/mL in young women to meet nascent tissue demands. This physiological signaling for increased iron acquisition closely follows the initial deviation of erythrocyte indices from their absolute plateau, which we observed at ~ 59 ng/mL. Therefore, values between 26 and 59 ng/mL represent a transitional state: iron stores are declining, triggering upregulated intestinal absorption (~ 51 ng/mL), but severe cellular derangements have not yet manifested (~ 26 ng/mL). Maintaining a first-trimester ferritin level ≥ 59 ng/mL thus provides a biologically protective reserve, ensuring physiological sufficiency before the massive expansion of maternal red blood cell mass and fetoplacental iron requirements in the second and third trimesters.

To further consolidate the threshold, we validated our FRL against TSAT. We employed a cutoff of 16%, which has a sensitivity of 20% and a specificity of 93%^21^ for identifying iron deficiency. Indeed, when tested against TSAT < 16%, the FRL demonstrated good specificity (76.2%) and an extremely high negative predictive value (95.8%), supporting its clinical utility as a rule-out boundary for iron deficiency. Therefore, we propose a two-step algorithm: (1) screen with ferritin; (2) for values 26–59 ng/mL, an additional step of measuring TSAT is needed. This strategy balances sensitivity and specificity and mitigates ferritin’s acute-phase confounding^22^.

From a clinical standpoint, our findings support a decisive shift away from long-standing ferritin cutoffs toward thresholds that are functionally and physiologically validated. Importantly, clinical decision limits must be distinguished from statistical reference limits. Ferritin values below 15 ng/mL—the red zone in Fig. 4—correspond to absolute iron deficiency, a state well documented in bone-marrow studies, and warrant prompt therapeutic replacement^21^. Between 15 ng/mL and the lower reference limit identified in our study (~ 26 ng/mL; orange zone), patients likely exhibit early-stage or functional iron deficiency and should receive moderate to high iron dose supplementation to prevent progression. The transitional yellow zone (26–59 ng/mL) represents an area of physiological uncertainty: inflammation-associated ferritin elevation in pregnancy complicates interpretation, and confirmatory markers such as TSAT should be obtained before decisions are made. By contrast, ferritin values ≥ 59 ng/mL (green zone) fall above the FRL where erythropoiesis is no longer iron-limited; this zone therefore represents a clinically reassuring status of iron sufficiency. Taken together, these physiologically grounded zones provide a more nuanced and actionable framework than traditional cutoffs and directly inform a modern strategy for early-pregnancy iron screening.

Guideline recommendations for iron screening in pregnancy remain inconsistent. Current US Preventive Services Task Force^23^ concluded that there was insufficient evidence for universal screening for iron deficiency in non-anemic pregnant adults. By contrast, the International Federation of Gynecology and Obstetrics recommends routine screening for iron deficiency in pregnancy using ferritin and/or TSAT whenever inflammation is suspected, regardless of anemia status. Recommendations from European Hematology Association stated that iron deficiency should be identified as early as possible in the first trimester^12^ but a threshold to determine iron deficiency is not defined. A 2025 expert consensus also suggested that a ferritin level of 50 ng/mL could be used as an initial screening threshold for iron deficiency in adults^24^, consistent with McCarthy’s recommended threshold of 60 ng/mL^5^. The finding of an FRL above the conventional cutoff aligns with evidence that current diagnostic thresholds may underestimate iron deficiency in pregnancy, suggested by many guidelines^25–27^. Randomized studies in non-anemic, low-ferritin patients (ferritin < 50 ng/mL) have demonstrated improved hematologic indices and reduced fatigue following oral or intravenous iron therapy^28^ and pregnant individuals with ferritin higher than 70 ng/mL do not develop iron deficiency or iron deficiency anemia later on^24^. Thus, while the conventional 30 ng/mL cutoff prioritizes sensitivity to detect iron deficiency anemia, our functionally derived limit may better reflect iron adequacy for optimal pregnancy outcomes. Therefore, the proposed approach aligns with latest guidelines to prevent third-trimester iron deficiency or iron deficiency anemia.

A unique challenge in deriving hematological FRLs in Southeast Asian populations is the high endemicity of genetic polymorphisms affecting globulins, particularly alpha- and beta-thalassemia. Thalassemia carriers typically present with microcytic, hypochromic erythrocytes (MCV < 80 fL, MCH < 27 pg) but maintain normal or elevated serum ferritin levels due to ineffective erythropoiesis and inappropriately elevated intestinal iron absorption^29^. The inclusion of these patients would severely confound the physiological modeling of IDE, as their erythrocyte indices are dictated by genetic mutations rather than iron availability. To mitigate this, our protocol mandated the strict exclusion of patients with hemoglobinopathies confirmed by hemoglobin electrophoresis (*n* = 6). Furthermore, to ensure no undiagnosed thalassemia carriers skewed the FRLs, we excluded an additional 40 patients who presented with unexplained microcytosis or hypochromia but lacked confirmatory normal electrophoresis results. By isolating our analysis to a cohort where alterations in red cell indices were driven exclusively by iron status, we safeguarded the integrity of our physiological models against regional genetic confounders.

Several limitations should be acknowledged when interpreting our findings. A primary limitation of our study is the prospective exclusion of anemic patients from the derivation cohort. As iron-deficient erythropoiesis (IDE) is a progressive continuum^17^, excluding patients with overt anemia inherently truncates the lower tail of the erythrocyte distribution. This truncation may introduce a degree of bias into the restricted cubic spline modeling by shifting the distribution upward, which can potentially elevate the exact mathematical location of the inflection point. Because serum ferritin was not measured in anemic patients as per our initial study design—which focused exclusively on screening for non-anemic iron deficiency—we were unable to include the full continuum of IDE in our final models. Nevertheless, we believe our derived FRL remains highly clinically relevant for defining optimal iron sufficiency. The remarkable concordance of our derived lower reference limit (~ 26 ng/mL) with recently published, physiologically derived thresholds for ID in pregnancy (25.8 µg/L)^19^, combined with the alignment of our ~ 59 ng/mL sufficiency plateau with the known physiological upregulation of systemic iron absorption (~ 51 ng/mL)^20^, suggests that our FRL accurately reflects the transition point where a healthy, non-anemic population enters functional vulnerability. Although the physiologic modeling using restricted cubic splines provides a biologically informed framework for defining ferritin thresholds, the analysis remains observational and cannot establish causality. Our cohort reflects a specific population of healthy, non-anemic pregnant Asian women in early gestation, and the derived thresholds—particularly the FRL—may not be directly generalizable to populations with different dietary patterns, genetic backgrounds,or inflammatory burdens. Furthermore, ferritin is an acute-phase reactant, and although individuals with overt infection were excluded, subclinical inflammation cannot be fully ruled out. While TSAT was used as an adjunct measure, other iron biomarkers such as soluble transferrin receptor, reticulocyte hemoglobin content, or hepcidin were not included. These markers could have provided additional mechanistic validation of the physiological zones and strengthened the interpretation of iron status under pregnancy-related inflammation. Moreover, the study did not include longitudinal follow-up to determine whether individuals classified in each ferritin zone subsequently developed iron deficiency or anemia later in pregnancy. However, the strong concordance between our physiologic ferritin thresholds and TSAT confirms that the proposed zone-based approach functions as a reliable rule-out and classification method. Finally, a critical limitation of this study—and of iron status evaluation globally—is the lack of international harmonization among commercial serum ferritin assays. Our functional reference limits (e.g., 59 ng/mL) were derived exclusively using the Roche Cobas analyzer. Because significant analytical bias exists between different assay methodologies, specific threshold values derived on one platform cannot be universally applied to others without risk of misclassification^14^. The reliance on platform-specific thresholds underscores the urgent need for international standardization of ferritin assays to ensure that physiologically derived clinical decision limits can be safely and uniformly implemented across diverse laboratory settings^30^.

In conclusion, establishing a ferritin FRL of 59 ng/mL enhances specificity in ruling out iron deficiency, reclassifies nearly 23% of first-trimester patients as iron-deficient—four times the prevalence detected by traditional criteria—and aligns more closely with transferrin saturation (TSAT < 16%) than with legacy ferritin thresholds. Equally important, ferritin screening can also help identify iron overload, a condition increasingly recognized for its association with adverse maternal outcomes such as gestational diabetes mellitus^31^ and potential oxidative stress–related effects on fetal growth and development^32^. Integrating ferritin measurement into routine first-trimester antenatal screening may therefore serve a dual purpose in preventing both deficiency and excess iron. Future studies should validate these threshold strategies across diverse obstetric populations and evaluate their impact on maternal metabolic health and neonatal outcomes.

## Data Availability

The data that support the findings of this study are available from the corresponding author (Huan Nguyen Pham; email: huanpmd@gmail.com) upon reasonable request.
